# Sessile Trichomes Play Major Roles in Prey Digestion and Absorption, While Stalked Trichomes Function in Prey Predation in *Byblis guehoi*

**DOI:** 10.3390/ijms24065305

**Published:** 2023-03-10

**Authors:** You-Xian Li, Alvin Chen, Wei-Ming Leu

**Affiliations:** 1 Ph.D. Program in Microbial Genomics, National Chung Hsing University and Academia Sinica, Taichung 40227, Taiwan; 2 Graduate Institute of Biotechnology, National Chung Hsing University, Taichung 40227, Taiwan

**Keywords:** carnivorous plant, *Byblis guehoi*, glandular trichome, sessile trichome, stalked trichome, FITC-BSA, plasmodesmata

## Abstract

Carnivorous plants in the genus *Byblis* obtain nutrients by secreting viscous glue drops and enzymes that trap and digest small organisms. Here, we used *B. guehoi* to test the long-held theory that different trichomes play different roles in carnivorous plants. In the leaves of *B. guehoi*, we observed a 1:2.5:14 ratio of long-stalked, short-stalked, and sessile trichomes. We demonstrated that the stalked trichomes play major roles in the production of glue droplets, while the sessile trichomes secrete digestive enzymes, namely proteases and phosphatases. In addition to absorbing digested small molecules via channels/transporters, several carnivorous plants employ a more efficient system: endocytosis of large protein molecules. By feeding *B. guehoi* fluorescein isothiocyanate-labeled bovine serum albumin (FITC-BSA) to monitor protein transport, we found that sessile trichomes exhibited more endocytosis than long- and short-stalked trichomes. The uptaken FITC-BSA was delivered to the neighboring short epidermal cells in the same row as the sessile trichomes, then to the underlying mesophyll cells; however, no signals were detected in the parallel rows of long epidermis cells. The FITC control could be taken up by sessile trichomes but not transported out. Our study shows that *B. guehoi* has developed a well-organized system to maximize its food supply, consisting of stalked trichomes for prey predation and sessile trichomes for prey digestion. Moreover, the finding that sessile trichomes transfer large, endocytosed protein molecules to the underlying mesophyll, and putatively to the vascular tissues, but not laterally to the terminally differentiated epidermis, indicates that the nutrient transport system has evolved to maximize efficiency.

## 1. Introduction

Carnivorous/insectivorous plants can hunt prey and digest them [1,2]. They utilize specialized traps to capture and consume prey, mainly arthropods, which allows them to thrive in nutrient-poor native environments. Generally, prey utilization involves four major steps: (1) attraction of prey through scent, nectar, or optical signals; (2) trapping and killing of prey by specialized trapping organs; (3) degradation of prey by digestive enzymes; (4) uptake of small molecule compounds into the plant [3]. Approximately 800 species of carnivorous plants have been described in five orders of flowering plants, namely Poales, Lamiales, Ericales, Oxalidales, and Caryophyllales [4,5]. The “traps” of carnivorous plants can be divided into five types according to their characteristics, namely adhesive traps (e.g., *Byblis* and *Drosera*), pitfall traps (e.g., *Nepenthes* and *Sarracenia*), snap traps (e.g., *Aldrovanda* and *Dionaea*), suction traps (e.g., *Utricularia*), and lobster pot traps (e.g., *Genlisea*). The most intriguing question about these fascinating plants is how they capture and digest prey [1,2,3,4,6,7].

The epidermis of carnivorous trap leaves usually bears groups of specialized cells called glands, also known as glandular trichomes, which secrete prominent droplets of mucilage. Many vascular plants have glandular trichomes, which secrete a wide variety of substances, including mucilage, resins, salts, nectar, aromatic compounds, and pathogenesis-related proteins [8,9,10,11,12]. However, the protease and phosphatase activities of carnivorous plant glands are key features that differentiate the trichomes of carnivorous plants from those of other vascular plants [13,14,15]. Multiple digestive enzymes have been identified in the mucilage of carnivorous plants, especially in the genera *Nepenthes* and *Cephalotus* [3,16,17,18,19].

Multiple types of glandular trichomes are commonly found in carnivorous plants. However, the different functions of glandular trichomes are not clearly understood for most carnivorous plants and are usually judged solely on the basis of their morphology and positioning [3]. *Byblis*, which is sometimes called the rainbow plant because of the attractive appearance of its mucilage-covered leaves under bright sunshine, is a typical example of a carnivorous plant that contains different types of glandular trichomes—sessile and stalked. *Byblis* use adhesive traps to catch prey with sticky glue. The heads of the stalked trichomes were reported to secrete viscous glue and may play a role in trapping prey, whereas the heads of sessile trichomes were described to be responsible for enzyme secretion and nutrient uptake. The above findings were first reported by Fenner [20] and Bruce [21] in *B. gigantea* using insects or coagulated egg-albumen as prey. By observing the fates of prey on sessile and stalked trichomes, the different roles of both trichomes were claimed ever since. However, owing to the lack of recording techniques, only descriptive results were presented and were cited by Lloyd [2]. Afterward, the various literature reiterated the differential roles of the two types of trichomes mainly based on the structural analysis, morphological or positional clues, or mucilage secretions of trichomes [3,4,22,23,24]. Although revealing illuminating information, no experiments were undertaken to re-examine such a known phenomenon. A paralleled experimental investigation and comparison of the roles of different trichomes is still lacking.

In addition to taking up nutrients via specific channels/transporters, several carnivorous plants take up large molecules via endocytosis. For example, Adlassnig et al. [25] demonstrated that fluorescein isothiocyanate-labeled bovine serum albumin (FITC-BSA) could be uptaken via endocytosis in various carnivorous plants with different evolutionary origins. Lichtscheidl [26] showed that cells of *Drosera* treated with FITC-BSA formed fluorescent endosomes, which pinched off from the plasma membrane, fused into larger aggregates, and accumulated in the bulk cytoplasm around the nucleus. Thereafter, likely through the plasmodesmata-mediated symplastic route, the fluorescent vesicles proceeded through cells within the tentacle stalk and reached the parenchymal cells. Endocytosis allows nutrients to be taken up more efficiently than simply via digestion and small molecule absorption. Nevertheless, *Byblis* was not included in previous endocytosis studies [25,26,27].

We aimed to provide visual/biochemical evidence for the contrasting roles of stalked versus sessile trichomes by paralleled examinations. Furthermore, we wondered if *Byblis* possesses endocytosis ability, and, if yes, what is the route for nutrient absorption and transport after prey capture in *Byblis*. To this end, we demonstrated through visual staining experiments that the stalked trichomes of *B. guehoi* secrete mainly pectin while the sessile trichomes secrete enzymes, namely proteases and phosphatases, indicating that they may play different roles in prey predation and digestion, respectively. Moreover, we found that the sessile trichomes also play a role in nutrient uptake via endocytosis. FITC-BSA could be taken up by the sessile trichomes within a short time, then be transported to neighboring short epidermal cells through a highly regulated symplastic route. FITC-BSA was then transported downward to the underlying mesophyll cells but never laterally to the long epidermal cells located parallel to the glandular trichomes. This directional intercellular transport suggests that *B. guehoi* has developed a highly organized and efficient nutrient absorption and transportation process enabling its survival in a nutrient-poor environment.

## 2. Results

### 2.1. Byblis Leaves Harbor Three Kinds of Glandular Trichomes

*B. guehoi* is a fast-growing carnivorous plant consisting mainly of stems and thin leaves aboveground, with sizes ranging from 10 cm to more than 50 cm in length (Figure S1A). Virtually all aboveground parts of *B. guehoi* are covered with mucilage-tipped stalked glands, which secrete glue drops and efficiently capture small insects that are passing by (Figure S1B).

**Figure 1:**
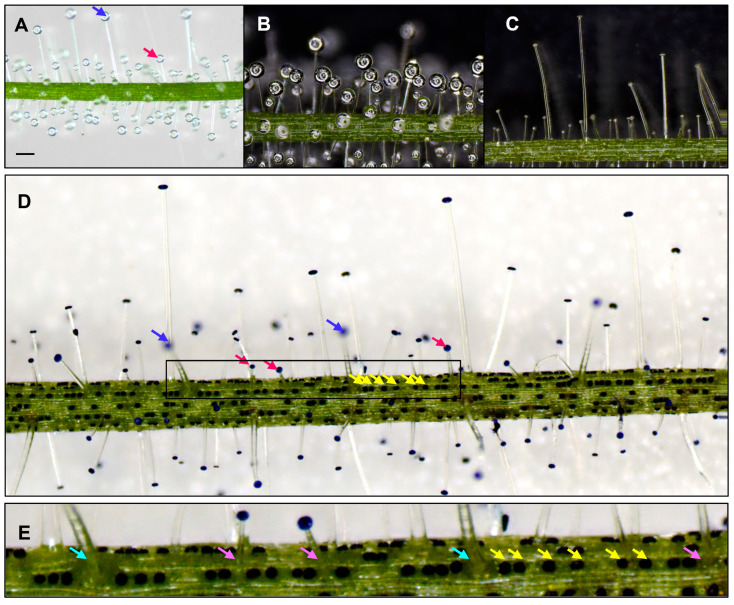
Types and characteristics of glandular trichomes of *B. guehoi.* (**A**) Stalked trichomes: long-stalked (blue arrows) and short-stalked (red arrows) forms. (**B**) Glue drops viewed by the extended depth of field processing (EDF). (**C**) Trichomes after a water wash viewed by EDF. (**D**) Three kinds of trichomes viewed after methylene blue staining. The blue, red, and yellow arrows indicate the long-stalked, short-stalked, and sessile trichomes, respectively. (**E**) Enlarged view of the outlined area in (**D**). The yellow, light red, and light blue arrows indicate the sessile trichomes, the basal cells of the short-stalked trichomes, and the basal cells of the long-stalked trichomes, respectively, in the “trichome row”. Scale bar = 0.5 μm.

*B. guehoi* has two types of glandular trichomes: sessile and stalked. Microscopic observation revealed that the stalked trichomes were distributed all over the surface of the stems and the filiform leaves and that there were two prominent populations: short-stalked trichomes, which were predominant, mixed with a few long-stalked trichomes (Figure 1A).

The head of each stalked trichome was covered with a droplet of sticky glue (Figure 1B). Washing the plants with water revealed that the glue drops were water soluble, and the stalked trichomes retained their shape after washing (Figure 1C), with the glue drops replenished within two days. The sessile trichomes, which do not have stalk cells, could be identified clearly after methylene blue staining (Figure 1D). On the leaves, the sessile trichomes were arranged in rows along with the short- and long-stalked trichomes, short epidermal cells, and guard cells (Figure 1E). Usually, these “trichome rows” were separated from each other by one or two “long epidermal cell rows”, which consisted mainly of long epidermal cells.

The average length of the long-stalked trichomes was 1.13 ± 0.41 mm (n = 134) on the upper surface of the leaf, significantly longer than that on the lower surface, which was 0.89 ± 0.23 mm (n = 159). In contrast, the short-stalked trichomes were slightly longer on the lower surface (0.44 ± 0.10 mm, n = 163) than on the upper surface (0.40 ± 0.11 mm, n = 196) (Figure S2B). The ratio of long-stalked, short-stalked, and sessile trichomes was about 1:2.5:14 by counting the half surface of fully-extended leaves (Figure S1A). The number of long-stalked trichomes was similar between the upper and the lower surfaces of the leaf. The lower leaf surface shows significantly higher densities in the short and sessile trichome than the upper surface, approximately 1.43 and 1.19 folds, respectively (Figure S2C). It is interesting to note that more insects were trapped on the lower surface than on the upper side of the leaves (Figure S1B).

### 2.2. Close-Up Observations of the Glandular Trichomes

**Figure 2:**
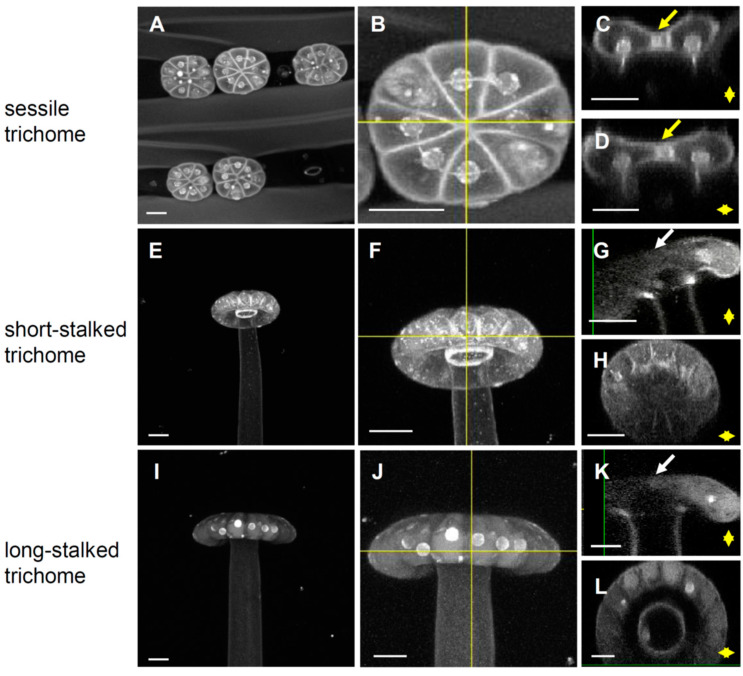
Confocal microscopic analysis of the fine structures of the glandular trichomes. Leaf segments were stained by propidium iodide (PI) and displayed as Z-projections. (**A**–**D**) Sessile trichomes; 56 Z slices were acquired every 0.8 μm with a stack height of 44.8 μm. (**E**–**H**) Short-stalked trichomes; 82 Z slices were acquired every 0.8 μm with a stack height of 65.6 μm. (**I**–**L**) Long-stalked trichomes; 99 Z slices were acquired every 0.8 μm with a stack height of 79.2 μm. (**C**,**G**,**K**) Longitudinal sections taken from the yellow vertical lines in (**B**,**F**,**J**), respectively. (**D**,**H**,**L**) Transverse sections taken from the yellow horizontal lines in (**B**,**F**,**J**), respectively. The yellow and white arrows indicate the presence and absence of surface indentation, respectively, in the middle of the head of trichomes. Scale bar = 20 μm.

To analyze the long-stalked, short-stalked, and sessile trichomes at the cellular level, leaf segments were incubated with propidium iodide (PI), which stains plant cell walls and nuclei, and then viewed under a confocal microscope. On fully expanded leaves, the heads of all glandular trichomes contained multiple cells. The heads of sessile trichomes always contained 8 cells (Figure 2A,B), while the heads of short-stalked and long-stalked trichomes contained 12 (Figure 2E,F,H) and 16 cells (Figure 2I,J,L), respectively. Nevertheless, the numbers of cells within the stalked trichomes varied, ranging from 12 to more than 30 cells in a preliminary analysis. Besides the difference in the number of cells, we noticed a shallow surface indentation located at the center of the sessile trichomes (Figure 2C,D), which was not present in the short-stalked or long-stalked trichomes (Figure 2G,K).

To further explore the subcellular structures of the glandular trichomes, we co-stained leaf segments with calcofluor-white (CFW) and auramine O (AuO), which preferentially bind to hydrophilic and hydrophobic compounds, respectively (Figure S3A–C). In all three types of trichomes, the CFW-stained substances appeared dispersed when viewed at the XY plane (Figure S3D,J,P), but there were prominent granules stained only by AuO (Figure S3E,K,Q). Z-scanning along the X or Y axis of individual trichomes (Figure S3G,M,S) revealed that the AuO-stained granules were located within the cytosol, whereas the CFW signals were located on the cell surface (Figure S3H,I,N,O,T,U). It would be very interesting to discover which substances are contained in the granules and what their functions are.

### 2.3. Differential Roles of Glandular Trichomes Are Suggested by the Different Compounds Secreted

**Figure 3:**
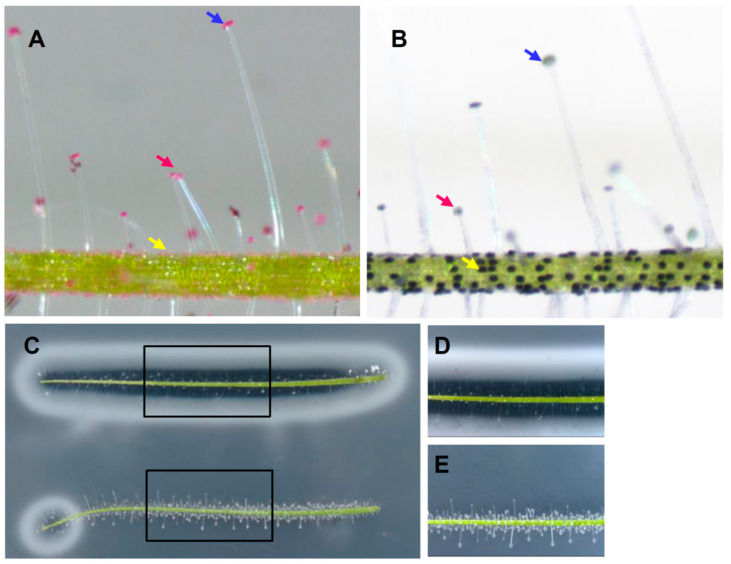
Secretion of enzymes from glandular trichomes. (**A**) Leaf segment stained by ruthenium red. (**B**) Leaf segment stained with the phosphatase substrate NBT/BCIT. (**C**) Leaf segments immersed in (**top**) or placed gently on (**bottom**) an agar plate containing 1% casein for 16 h. (**C**–**E**) The blue, red, and yellow arrows indicate the long-stalked, short-stalked, and sessile trichomes, respectively.

We were curious as to whether the different forms of trichomes play different roles in predation. As the glue drops are theoretically mainly composed of pectin, we stained trichomes with ruthenium red, which preferentially stains acid polysaccharides. As shown by Figure 3A, all the long-stalked and short-stalked trichomes accumulated abundant pink-stained pectin within their heads. In contrast, the sessile trichomes exhibited relatively low ruthenium red staining.

In addition to pectin, the glandular trichomes in carnivorous plants also theoretically secrete abundant enzymes including proteases and phosphatases. When leaves were incubated with the nitro blue tetrazolium (NBT)/5-bromo-4-chloro-3-indolyl phosphate (BCIP), which is used to detect phosphatase activity, the sessile trichomes exhibited strong phosphatase activities; however, little activity was detected in the short-stalked and long-stalked trichomes (Figure 3B). To examine the protease activity, we performed assays using casein-containing plates. A clear zone, indicating substrate digestion by proteases, surrounding the leaf segment was observed only when the leaf segment was embedded within agar, but not when it was gently placed on top of the agar (Figure 3C). A close-up picture showed that the stalked trichomes hindered the contact between the sessile trichomes and the agar when the leaf segment was placed on top of the agar (Figure 3E) but not when the leaf segment was fully immersed (Figure 3D). The protease activity of the sessile trichomes was also assayed using plates containing 1% skim milk (Figure S4A–C) or 1% gelatin (Figure S4D–F), and the phosphatase activity was assayed using a plate containing NBT/BCIP (Figure S4G–I). In all these assays, strong enzyme activities could be detected only when the sessile trichomes on the leaf segment were in contact with the substrates in plates.

Taken together, these results indicate that the sessile trichomes, not the short-stalked or long-stalked trichomes, play a major role in the secretion of phosphatases and proteases. We conclude that the short-stalked and long-stalked trichomes a play major role in prey capture by secreting pectin, while the sessile trichomes play a major role in prey digestion by secreting enzymes.

### 2.4. Differential Roles of Glandular Trichomes Are Suggested by the Different Rates of Nutrient Uptake

**Figure 4:**
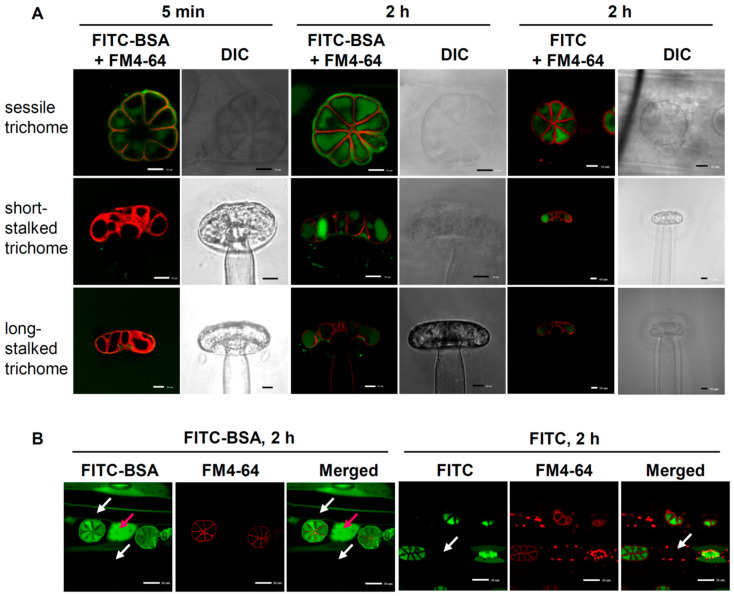
Uptake of FITC-BSA by the glandular trichomes. (**A**) Leaf segments were first incubated with FM4-64 and then incubated with FITC-BSA for 5 min (left panel) or 2 h (middle panel), or with FITC for 2 h (right panel). A single slice without Z-stacking is shown. (**B**) Same as (**A**) but the sessile trichomes and several neighboring cells were observed together. Red and white arrows indicate cells with and without FITC signals, respectively. Scale bar for (**A**), 10 μm; for (**B**), 30 μm.

Carnivorous plants absorb digested small molecule nutrients through transporters or channels, but the existence of a more efficient endocytosis pathway has been demonstrated in several carnivorous plants. We examined the possibility that *B. guehoi* also uses endocytosis for digestion by incubating the leaf segments of *B. guehoi* with FITC-BSA, which traces endocytosis, and FM4-64, which stains the plasma membrane. After 5 min of incubation, faint FITC-BSA signals surrounded by FM4-64 signals were observed in the sessile trichomes but not in the short-stalked or long-stalked trichomes (Figure 4A, left panel). Over a prolonged incubation (2 h), FITC-BSA was taken up continuously into the sessile trichomes and strong signals were observed. Faint FITC-BSA signals were also observed in the short-stalked and long-stalked trichomes (Figure 4A, middle panel). This difference in the rate of absorption suggests that the sessile trichomes play a major role in the endocytosis of large molecules.

### 2.5. Route of Intercellular Transport of Nutrients after Uptake by Sessile Trichomes

We next asked what the fate of nutrients taken up by the sessile trichomes in *B. guehoi* are. After 2 h of incubation, abundant FITC-BSA signals were observed in the sessile trichomes and also in the neighboring short epidermal cells in the same row. Surprisingly, no FITC-BSA signals were found in the long epidermal cells in parallel rows, suggesting the lack of both direct absorption and indirect transport of FITC-BSA from sessile trichomes to these cells (Figure 4B, left panel). To rule out the possibility that the FITC-BSA molecules were actually cleaved by proteases secreted from *B. guehoi* allowing the FITC to be imported into the sessile trichomes via transporters or channels, we used FITC molecules alone as a control. We observed that, similar to FITC-BSA, FITC molecules were taken up by all three kinds of trichomes (Figure 4A, right panel). Nevertheless, in contrast to FITC-BSA, no FITC molecules alone were transported to the neighboring short epidermal cells after being absorbed by sessile trichomes (Figure 4B, right panel). This result suggests that the transport of large molecules taken up by the sessile trichomes to the neighboring cells is a well-controlled process.

**Figure 5:**
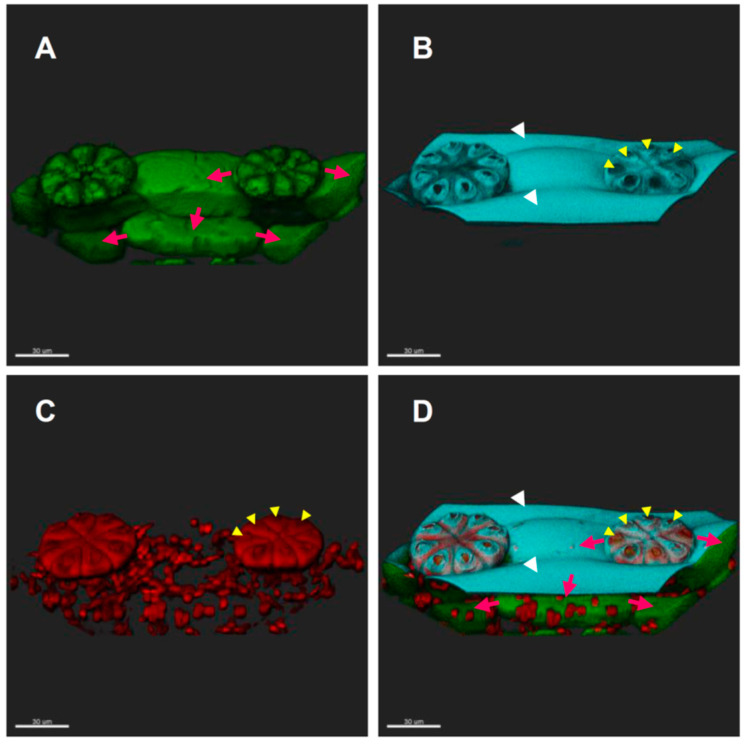
Intercellular transport of FITC-BSA after being taken up by the sessile trichomes. Leaf segments were co-incubated with FM4-64 and CFW, then incubated with FITC-BSA for 2 h; 86 Z slices were acquired by a confocal microscope every 0.5 μm, covering a total height of 43 μm. Imaris Viewer was used for 3D reconstruction. (**A**) FITC-BSA; (**B**) CFW; (**C**) FM4-64; (**D**) merged. Red arrows indicate FITC-BSA signals in cells neighboring or underneath the sessile trichomes. White arrowheads indicate neighboring rows of long epidermal cells without FITC-BSA signals. Yellow arrowheads indicate pores on top of every cell in the sessile trichome. Scale bar = 30 μm.

To examine whether FITC-BSA can be delivered to the underlying mesophyll cells, we incubated the leaf segments with CFW and FM4-64, then with FITC-BSA for 2 h. Imaris Viewer was applied to view the cell layers under the epidermis. As shown in Figure 5A and Movie S1, the incorporated FITC-BSA signals were located in the sessile trichomes, the neighboring short epidermal cells between trichomes, and also in the mesophyll cells directly beneath them. However, although the long epidermal cells located in parallel with the row containing trichomes could be identified by CFW staining (Figure 5B), no FITC-BSA signals were found in them (Figure 5A). This observation suggests that the nutrients, after being taken up by the sessile trichomes, were transported via the symplastic route to the neighboring short epidermal cells, and then downwards to the underlying mesophyll cells; however, they were not transported to the long epidermal cells located in the parallel rows.

For semi-quantification of the FITC-BSA signals, the images in Figure 5A were rotated to orient the sessile trichomes horizontally, then FITC-BSA signals were quantified using the Image J program (Figure S5). This analysis revealed that the FITC-BSA signals were the most abundant in the sessile trichomes where FITC-BSA was absorbed, as indicated by the white color representing the highest FITC-BSA signal intensity. Based on the signal intensities, we speculated that FITC-BSA was then transported laterally to the neighboring short epidermal cells as shown by the yellow/orange color in Figure S5, then vertically downwards to cells beneath the neighboring cells as shown by the purple/blue color. The cells directly beneath the trichomes also contain FITC-BSA signals; however, these signals are relatively weak, making it impossible to determine their source. (Figure S5).

Interestingly, a distinctive pore was found on top of every cell in the sessile trichome; this pore was particularly obvious when stained with CFW (Figure 5B) followed by FM4-64 (Figure 5C), but no pore was visible when leaf segments were incubated with FITC-BSA (Figure 5A). This observation suggests that there may be a cuticular discontinuity of the cell wall. We suspect that these pores may a play role in the entrance of FITC-BSA into the sessile trichomes.

### 2.6. Pores on the Sessile Trichomes Allow the Entrance of FITC-BSA

**Figure 6:**
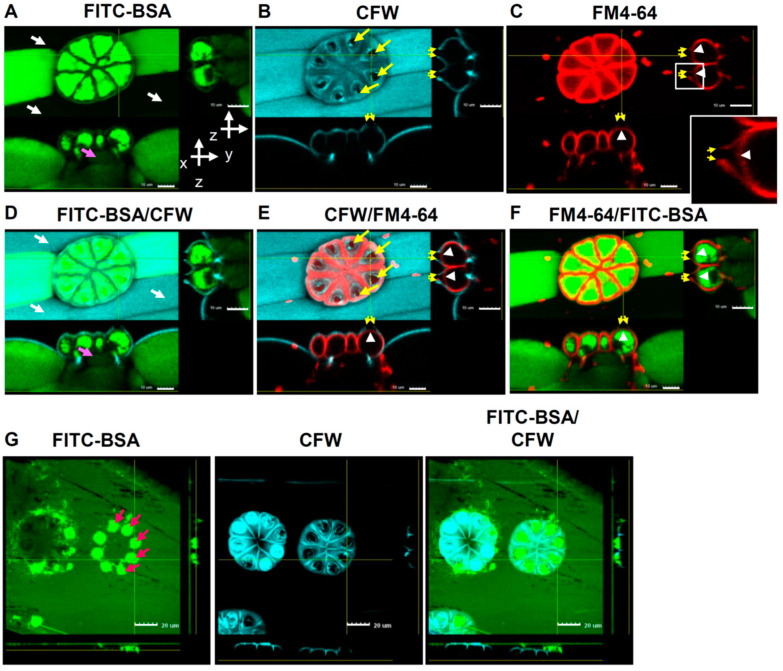
Uptake of FITC-BSA via pores on each head cell of the sessile trichomes. Leaf segments were first co-incubated with FM4-64 and CFW, then incubated with FITC-BSA for 2 h (**A**–**F**) or 5 min (**G**) and displayed as z-projections to observe the staining. For A-F, 25 Z slices were acquired every 0.5 μm covering a total height of 12.5 μm. For G, 20 Z slices were acquired every 0.9 μm covering a total height of 18 μm. The side and bottom images for each picture show the longitudinal and the transverse sections, respectively, taken from sections indicated by the yellow lines. White and pink arrows indicate no and weak signals of FITC-BSA, respectively. Yellow arrows indicate pores on cells. Small yellow arrows and white arrowheads indicate volcanic craters and round barriers detected by CFW/FM4-64 and FM4-64, respectively, around pores of the sessile trichomes. The inset in (**C**) is an enlarged image of the boxed area that shows the intactness of the plasma membrane detected by FM4-64. Red arrows indicate the FITC-BSA that rapidly accumulated on pores. Scale bar for (**A**–**F**), 10 μm; for (**G**), 20 μm.

To view the pores on the sessile trichomes more clearly, confocal Z-scanning was performed on leaf segments stained with CFW and FM4-64 and then incubated with FITC-BSA for 5 min or 2 h. Pictures at the X/Z and Y/Z planes revealed a clear discontinuity, with a shape similar to that of a volcanic crater, on the cell surface when leaves were stained with CFW (Figure 6B) or FM4-64 (Figure 6C), but not with FITC-BSA (Figure 6A). FM4-64 staining also revealed a round-shaped continuous signal below the pore, which likely represents the cell membrane (Figure 6C, inset) that surrounded the FITC-BSA signals within the cytosol (Figure 6F). Merged images showed that discontinuity was in the cell wall and that the cell membrane was intact (Figure 6E,F). Moreover, the FITC-BSA signals were absent in the parallel long epidermal cells and, interestingly, they were also very weak in the neck cells of the sessile trichomes viewed at the X/Z plane (Figure 6A,D).

Next, we stained the cell walls with AuO, which preferentially binds hydrophobic compounds and is often used to stain cutin, to examine the region of discontinuity in the cell wall. To avoid interference from the prominent granules stained by AuO within the cytosol (Figure S3), we generated confocal Z-projections with thin stacks. Clear pores were observed by AuO staining (Figure S6), similar to what was observed with CFW staining. This observation suggests that the pores on sessile trichomes lack both hydrophilic and hydrophobic compounds.

We were curious about what role the discontinuity of the cell wall plays in endocytosis. Using short-term labeling (5 min), we found that FITC-BSA signals were densely localized around the CFW-free region (Figure 6G). This observation suggests that the discontinuous region in the cell wall plays an important role in the uptake of FITC-BSA.

## 3. Discussion

### 3.1. Different Glandular Trichomes Play Different Roles in B. guehoi and Other Carnivorous Plants

The long- and short-stalked trichomes, which secrete abundant adhesive glue drops, capture small passing insects. The insects are likely to die from suffocation due to the clogging of their tracheae. Recently, the long-held theory that the traps of *Byblis* are nonmotile was first officially disproved by Allan [24] and further investigated by Poppinga et al. [28] at a cellular level. Through detailed analysis, the authors showed that even though slow in motion, the single-celled stalked trichomes of *B. gigantea* do react to chemical treatment by moving. These movements, which consist of twisting and kinking of the cell walls, were actuated by a continuous loss of water from the stalk cell, causing the stalked trichomes to eventually collapse and bend the captured prey toward the surface of the leaf.

Consistent with the observation in *B. gigantea*, we demonstrated that in *B. guehoi,* the stalked trichomes secrete sticky glue abundantly and, therefore, play a role mainly in prey predation. We also demonstrated that only the surface-located sessile trichomes secrete abundant digestion enzymes and absorb large nutrient molecules efficiently via endocytosis. Therefore, the stalked and sessile trichomes play different and complementary roles to supply *B. guehoi* with precious nutrients.

Similarly, distinctive but cooperative roles were found in the hierarchically structured traps of *Roridular gorgonias*, a carnivorous plant that secretes resinous glue drops. Without sessile glands and digestive enzymes, *Roridula* plants need the assistance of resident bugs to break down prey and supply them with nitrogen-rich feces. Nevertheless, *Roridula* has evolved an exquisite capture system in which the long, flexible trichomes mediate the initial trapping, while the short, stiff but very sticky trichomes are responsible for the long-term adherence of prey [29]. A high trapping efficiency was evidenced by the large numbers and varieties of insects trapped and the thriving of *Roridula* plants in its habitat.

### 3.2. Incorporation of FITC-BSA into Sessile Cells Is Mediated by Endocytosis

FM4-64 is a vital stain that fluoresces only in living cells. Cells cannot be fixed then stained nor stained then fixed. Therefore, the pictures in Figure 4, Figure 5, Figure 6 and Figure S5 of leaves stained by FM4-64 demonstrated that all cells studied were alive. Moreover, as FM4-64 is an endocytosis marker, which only generates a strong signal when it is inserted into the outer leaflet of the plasma membrane and then passed onto intracellular membrane compartments by endocytosis [30], the lack of signals in cells in rows parallel to those containing sessile trichomes indicates that they all lack endocytosis (Figure 5C). Only the eight cells in the sessile trichomes displayed strong endocytosis activities.

### 3.3. Nutrients Are Absorbed via Pores Located on the Sessile Trichomes of B. guehoi

Sessile trichomes are thought to be the sites for nutrient uptake in addition to secreting extracellular enzymes; however, this theory has only been supported by indirect evidence so far: specific cells in carnivorous plants are permeable to methylene blue [25,27,31]. Here, we observed that after incubating leaf segments with FITC-BSA for 5 min, abundant signals accumulated around pores located on the eight cells of the sessile trichomes (Figure 6G). These pores overlapped with the areas that were not stained by either CFW or AuO (Figure 6 and Figure S5), indicating that the pores may be caused by a discontinuity of cuticles, which have been described as cuticular pores in the literature [3,25,26,31,32]. However, the pores were also clearly visible as a cavity after CFW staining (Figure 6B), suggesting a deficiency of hydrophilic components such as cellulose and pectin, which are preferentially stained by CFW [33,34]. It is possible that the major obstacles for endocytosis in plants, namely the cuticle on the cell surface and cellulose within the cell wall, are both defective in the pores of sessile trichomes, allowing the plasma membrane to come into contact with the large protein molecules and endocytose them. The physical and biochemical attributes of the pores may help *Byblis* to locally accumulate large molecules and, therefore, enhance their endocytosis.

### 3.4. Suspected Polar Distributions of Plasmodesmata in the “Trichome ROW” of Epidermal Cells

We observed that FITC-BSA, but not FITC molecules alone, were transported to the adjacent neighboring cells of sessile trichomes (Figure 4A), suggesting the involvement of a selective mechanism, most likely mediated by plasmodesmata. Moreover, FITC-BSA absorbed by sessile trichomes was sent downwards to the underlying mesophyll cells but not laterally to the parallel rows of long epidermal cells (Figure 4, Figure 5 and Figure 6). We were curious about this phenomenon.

Leaf epidermal cells are known to originate from the L1 cell layer in primordia, and these cells expand largely via anticlinal cell divisions, thereby giving rise to a single discrete cell layer [35]. The efficient movement of FITC-BSA from the sessile trichomes to the neighboring short epidermal cells suggests that cells within the same “trichome row” may arise from a single cell lineage in which the primary plasmodesmata were established during cell division. Conversely, no FITC-BSA signals were detected in the “long epidermal cell row” right beside the “trichome row”, which indicates that these cell lineages are independent. Moreover, it is likely that no secondary plasmodesmata were formed between different rows of epidermal cells either, making the epidermal rows in *Byblis* leaf symplasmically isolated [36]. By contrast, the “trichome row” may establish abundant secondary plasmodesmata with the mesophyll cells underneath and, therefore, move the uptaken food downwards to the vascular bundles efficiently. It would be interesting to examine whether there is a polar distribution of plasmodesmata within the “trichome row” of epidermal cells in the *B. guehoi* leaf.

## 4. Materials and Methods

### 4.1. Source of B. guehoi and Plant Cultivation

Seeds of *B. guehoi* were purchased from the Best Carnivorous Plants store (http://www.bestcarnivorousplants.net (accessed on 1 January 2015)). Plants were germinated in a Petri dish and then planted in 2-inch pots with a 1:1 mixture of Akadama soil (red ball earth, particle size from 2–3 mm, Kanuma City, Japan): Perlite (Nan Hai Gardening Co., Xinbei City, Taiwan). Plants were grown in a windless environment at 25 °C with a humidity level from 60–80% and a 16 h light/8 h dark cycle under white light (photosynthetic photon flux density 110 μmol m^−2^ s^−1^). A small amount of peat soil (Jiffy substrate, Jiffy Growing Solutions, Zwijndrecht, The Netherlands) was used as a supplementary fertilizer.

### 4.2. Measurement of Lengths and Densities of Glandular Trichomes

The fully extended leaves, the seventh to ninth counted from the top of plants, were stained with 0.1% (*w*/*v*) methylene blue (Sigma-Aldrich, St. Louis, MO, USA) before photography. Leaves, aligned with upper- and lower-leaf sides orientated at the correct positions, were pictured from the basal to the apical parts of the leaves. In total, nine leaves from three plants were fully analyzed using Fuji/ImageJ.

### 4.3. Histochemical Staining and Light Microscopy

All leaf segments were washed thoroughly with tap water to remove the sticky glue from the surface before staining. Ruthenium red is used on plant materials for staining pectin, mucilage, and gum [37]. Leaf segments were incubated in a 0.01% (*w*/*v*) ruthenium red (Sigma-Aldrich, St. Louis, MO, USA) solution for 10 min to stain pectic acid. Moreover, as methylene blue is not able to penetrate intact cuticles [2], leaf segments were stained with a 0.1% (*w*/*v*) solution of methylene blue (Sigma-Aldrich, St. Louis, MO, USA) for 10 min to detect cuticle pores. Leaf segments were washed twice to remove excess dye before being observed under a dissection microscope (Nikon, SMZ745T, Tokyo, Japan).

### 4.4. Confocal Laser Scanning Microscopy and Image Acquisition

To prepare the leaf segments for staining, they were thoroughly cleaned with tap water to remove sticky glue. All dyes were dissolved in water and vital stains were employed so that cells could be kept alive for the endocytosis experiments. Leaf segments were soaked in a 40 μg/mL propidium iodide (PI, Molecular Probes, OR, USA) solution for 30 min to stain the plant cell wall and nucleus [38]. To further distinguish the cell components, Calcofluor-white (CFW) and Auramine O (AuO), which preferentially bind hydrophilic and hydrophobic compounds, respectively, were used [39]. Sequential staining was performed by staining with CFW (100 μg/mL) for 30 min, rinsing with water three times, then staining with AuO (100 μg/mL) for 30 min. After staining, leaf segments were rinsed three times with water to remove the excess dye.

After PI staining, a confocal microscope (Olympus FV1000, Tokyo, Japan) with 543 nm excitation/555–655 nm emission was used for analysis. For the CFW/AuO experiment, an FV1000 (Figure S3) or FV3000 confocal microscope (Figure S6) was used. In both experiments, 405 nm excitation/430–480 nm emission was used to view CFW, and 488 nm excitation/505–530 nm emission was used to view AuO. Z-projections were generated using the FV-ASW 3.0 v.1 and FV31S-SW v.2.2.1 software for FV1000 and FV3000, respectively.

### 4.5. Enzyme Activity Assays

For plate activity assays, one leaf/stem segment was laid on unsolidified medium containing the substrates. After solidification, another leaf/stem segment was laid gently on the surface. For the detection of phosphatase activity, 338 μg/mL nitro blue tetrazolium (NBT, Biosynth, Staad, Switzerland) and 175 μg/mL 5-bromo-4-chloro-3-indolyl phosphate (BCIP, Merck & Co., Inc., Rahway, NJ, USA) were added into 1% dissolved agar (Hispanagar, Burgos, Spain) at about 55 °C as substrates. Plates were incubated at 28 °C from 3–6 h until a purple precipitate formed. For the detection of protease activity, 1% casein (Sigma-Aldrich, St. Louis, MO, USA), 1% (*w*/*v*) skim milk, or 1% gelatin (Sigma-Aldrich, St. Louis, MO, USA) was added into 1% dissolved agar. Plates were incubated at 28 °C from 16–24 h until a clear zone formed.

### 4.6. Nutrient Uptake Assays

Fluorescein isothiocyanate (FITC)-conjugated BSA (FITC-BSA, Sigma-Aldrich, St. Louis, MO, USA) was used to trace the endocytosis process. Leaf segments were washed thoroughly with tap water to remove the sticky glue from the surface before staining. Leaf segments were co-stained with the vital stains FM4-64 (Thermo Fisher, Waltham, MA, USA) and CFW to mark the plasma membrane and cell wall, respectively, of sessile trichomes. Leaf segments were soaked in a solution containing 10 μg/mL FM4-64 and 10 μg/mL CFW, placed under a vacuum for 10 min, then incubated in the stain solution for 50 min. Next, leaf segments were rinsed with water and then incubated with solutions containing 100 μg/mL FITC-BSA or 90 μg/mL FITC for 5 min or 2 h before the reaction was stopped by washing.

A confocal microscope (Olympus FV3000, Tokyo, Japan) was used with 561 nm excitation/680–780 nm emission to view FM4-64, and 488 nm excitation/500–540 nm emission to view FITC-BSA and FITC. Z-projections were generated using the FV31S-SW v.1 software. IMARIS viewer (BITPLANE, http://www.bitplane.com/, version: x64 9.3.1) was employed for a 3D reconstruction to better present the relative position of cells and to rotate images for semi-quantitative analysis of the FITC-BSA signals using ImageJ.

## 5. Conclusions

This study provides evidence that in *B. guehoi*, the long- and short-stalked trichomes play major roles in the secretion of a sticky pectin-containing substance, which is responsible for prey capture. Sessile trichomes, however, play crucial roles in prey absorption, not only by secreting various digestive enzymes but also by absorbing large protein molecules efficiently. In the sessile trichomes, the nutrients absorbed are not transported laterally to parallel rows of the long epidermal cells; instead, they are transported to underlying mesophyll cells, where they are putatively delivered to the vascular bundles. Our results suggest that *B. guehoi* has developed a highly organized and efficient nutrient transportation process enabling survival in a nutrient-poor environment.

## Data Availability

Not applicable.

## Supplementary Materials

The following supporting information can be downloaded at: https://www.mdpi.com/article/10.3390/ijms24065305/s1.
