# Supplementary material for: Sessile Trichomes Play Major Roles in Prey Digestion and Absorption, While Stalked Trichomes Function in Prey Predation in Byblis guehoi
Source: Int J Mol Sci. 2023 Mar 10;24(6):5305. doi: 10.3390/ijms24065305 (PMC10048915; doi:10.3390/ijms24065305)
Supplement: Supplementary file 1 [file ijms-24-05305-s001.zip › ijms-2252264- final supp.pdf]

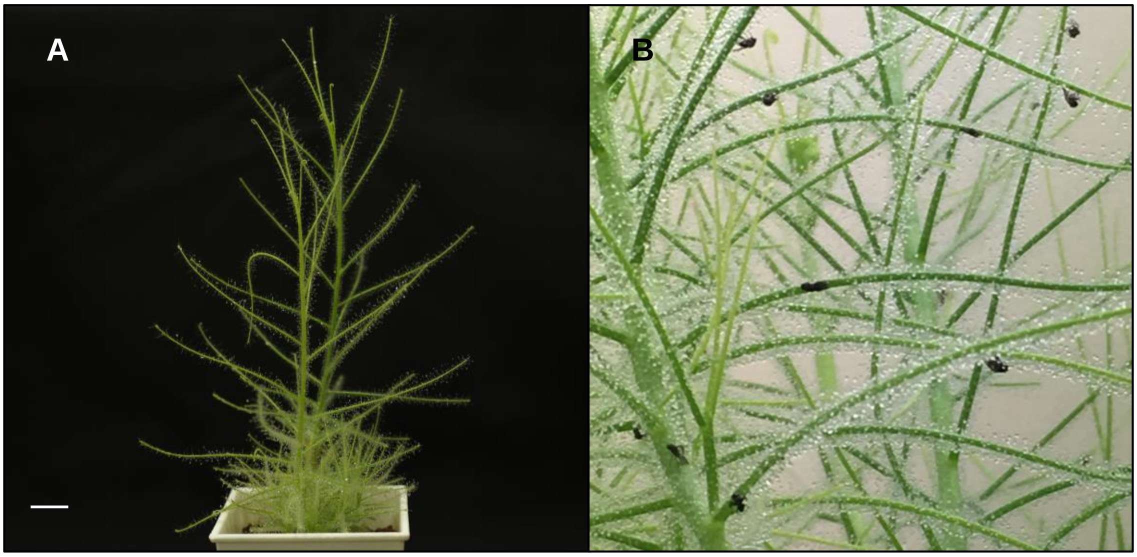

Figure S1. Phenotypic characteristics of *B. guehoi.* (A) Young seedling. (B) Leaves with trapped insects. Scale bar = 1 cm.

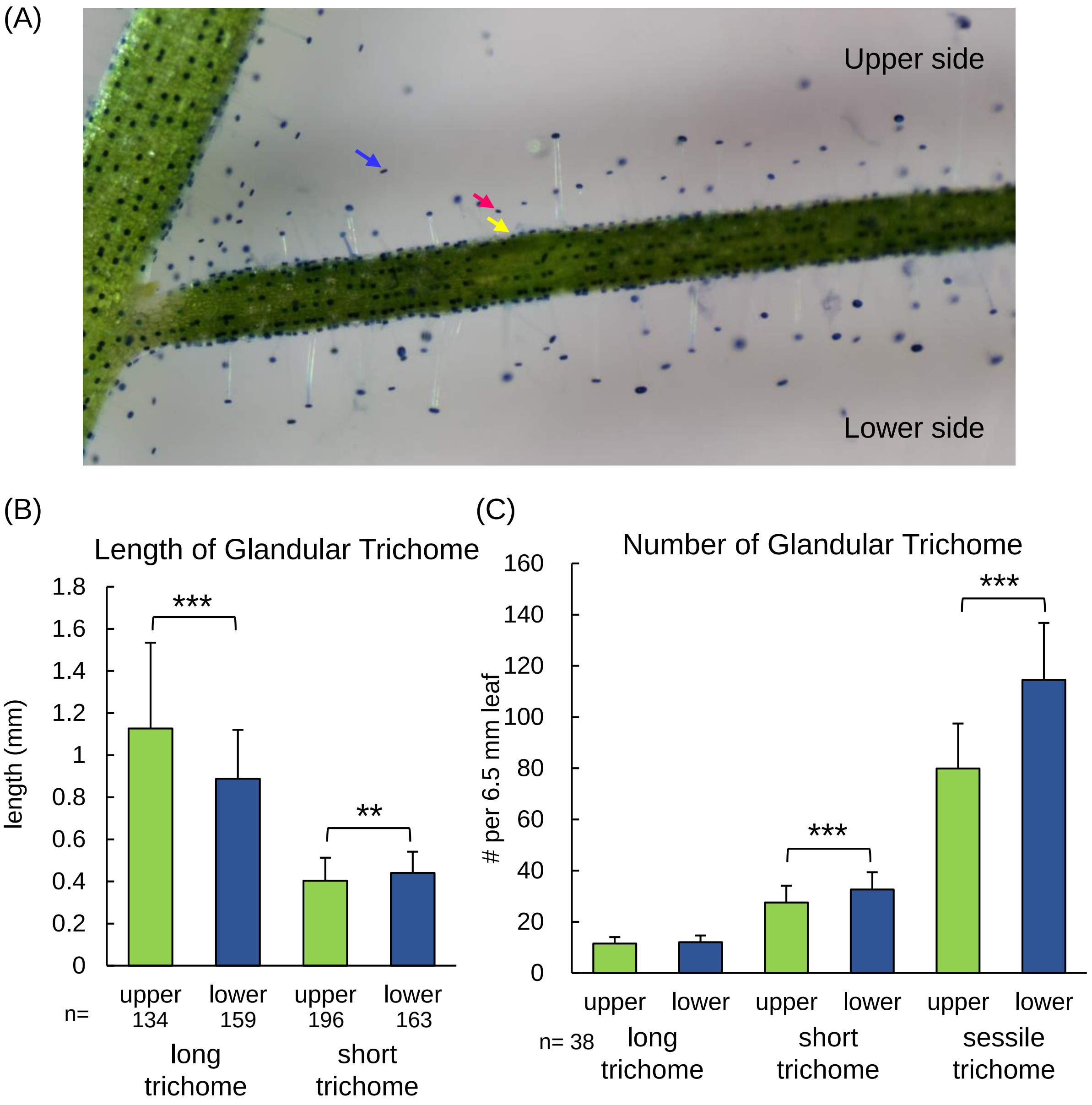

Figure S2. The length and number of the long-stalked, short-stalked, and sessile trichomes distributed on the upper and lower side of the leaf. (A) One fully extended leaf (the 7th leaf counted from the top) of *Byblis* was stained with 0.1% (w/v) methylene blue and with the upper- and lower-leaf sides orientated in the correct position. The blue, red, and yellow arrows indicate the long-stalked, short-stalked, and sessile trichomes, respectively. (B) The length of the trichome was quantified by Fuji/ImageJ. The long trichomes were arbitrarily defined by a length over 0.6 mm. (C) For the long-stalked and short-stalked trichomes, all trichomes on the upper and the lower sides were counted. For the sessile trichomes, only the top and bottom rows of trichomes were counted. Statistical analysis was performed using the Student's unpaired t-test for the length of trichomes and paired t-test for the number of trichomes. **p<0.01, and ***p<0.001.

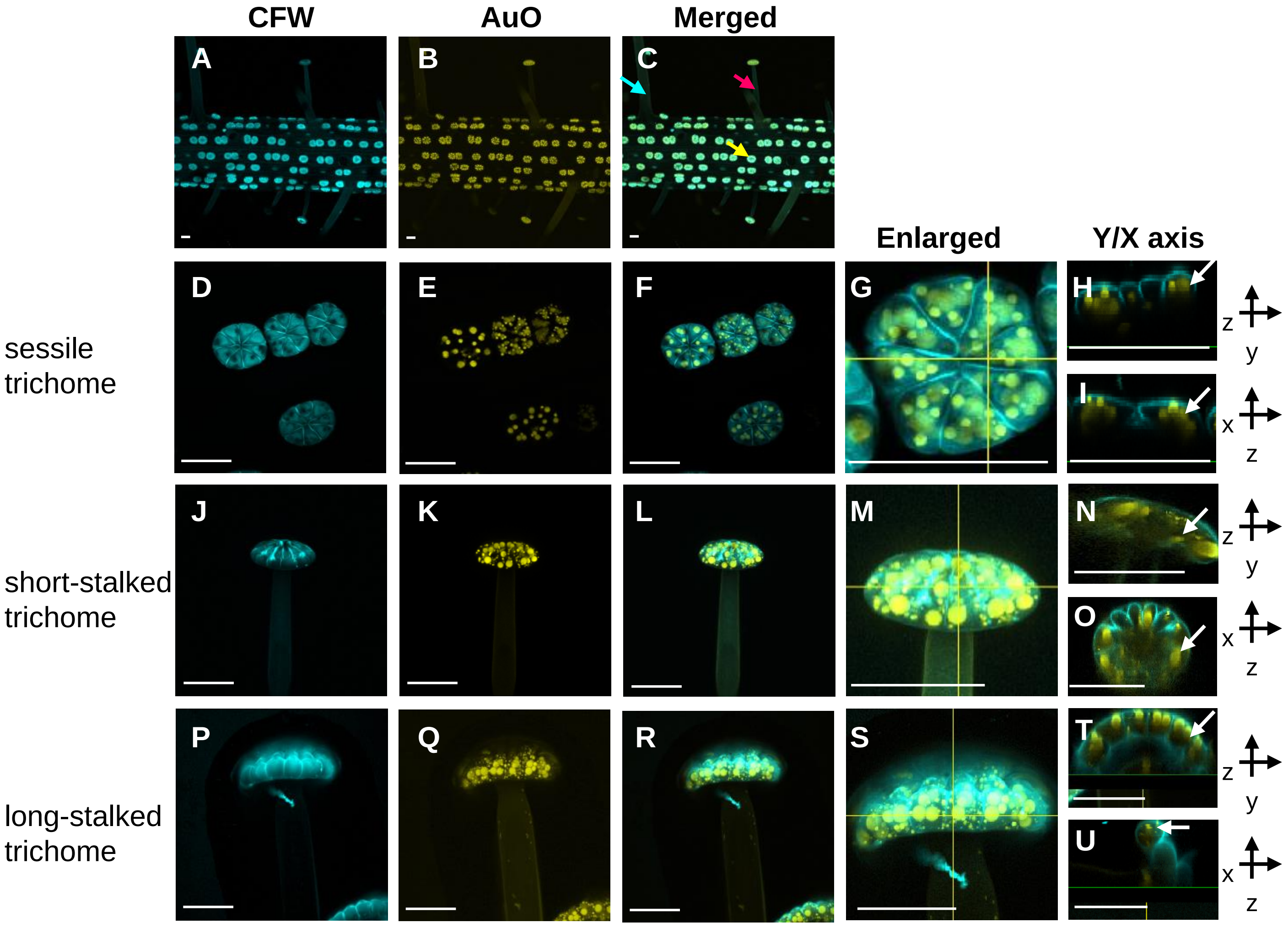

Figure S3. Confocal microscopic analysis of the fine structures within the glandular trichomes. Leaf segments were co-stained with calcoflour white (CFW) and auramine O (AuO) and then displayed as z-projections. (A, D, J, P), CFW channel; (B, E, K, Q), AuO channel; (C, F, L, R), merged; (G, M, S), merged then enlarged. (H, N, T), Longitudinal sections of G, M, and S, respectively. (I, O, U), Transverse sections of G, M, and S, respectively. The blue, red, and yellow arrows indicate the long-stalked, short-stalked, and sessile trichomes, respectively. The white arrows indicate the cytosol-located granule-like structures stained only by AuO. Scale bar = 50 μm.

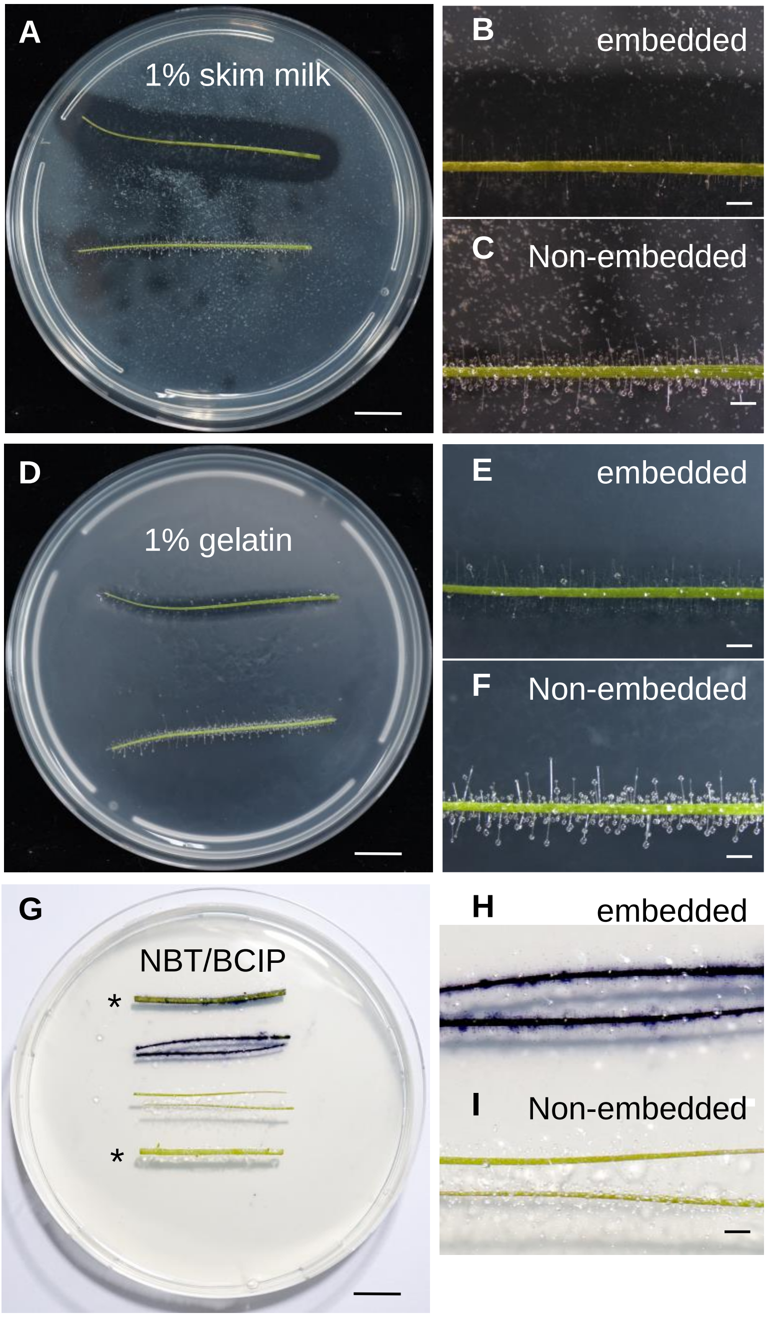

Figure S4. Enzyme activities detected in the sessile trichomes. (A-F) Protease activities detected after incubation for 16 h using 1% casein or 1% gelatin as substrate. (G-I) Phosphatase activity detected after 6 h using NBT/BCIP as substrate. (B), (E), and (H), leaf and stem (*) segments embedded within agar (embedded). (C), (F), and (I), Leaf and stem (*) segments that remained intact on top of agar (non-embedded). Scale bar for (A), (D), and (G), 1 cm; for others, 1 mm.

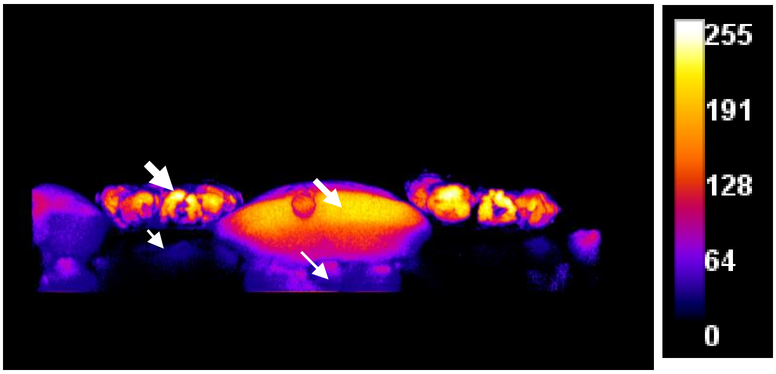

Figure S5. Semi-quantification of FITC-BSA taken up by the sessile trichomes. The same picture from Figure 5 was angle-adjusted and semi-quantified using ImageJ. White arrows from thick to thin reflect the signal intensities of FITC-BSA from strong to weak.

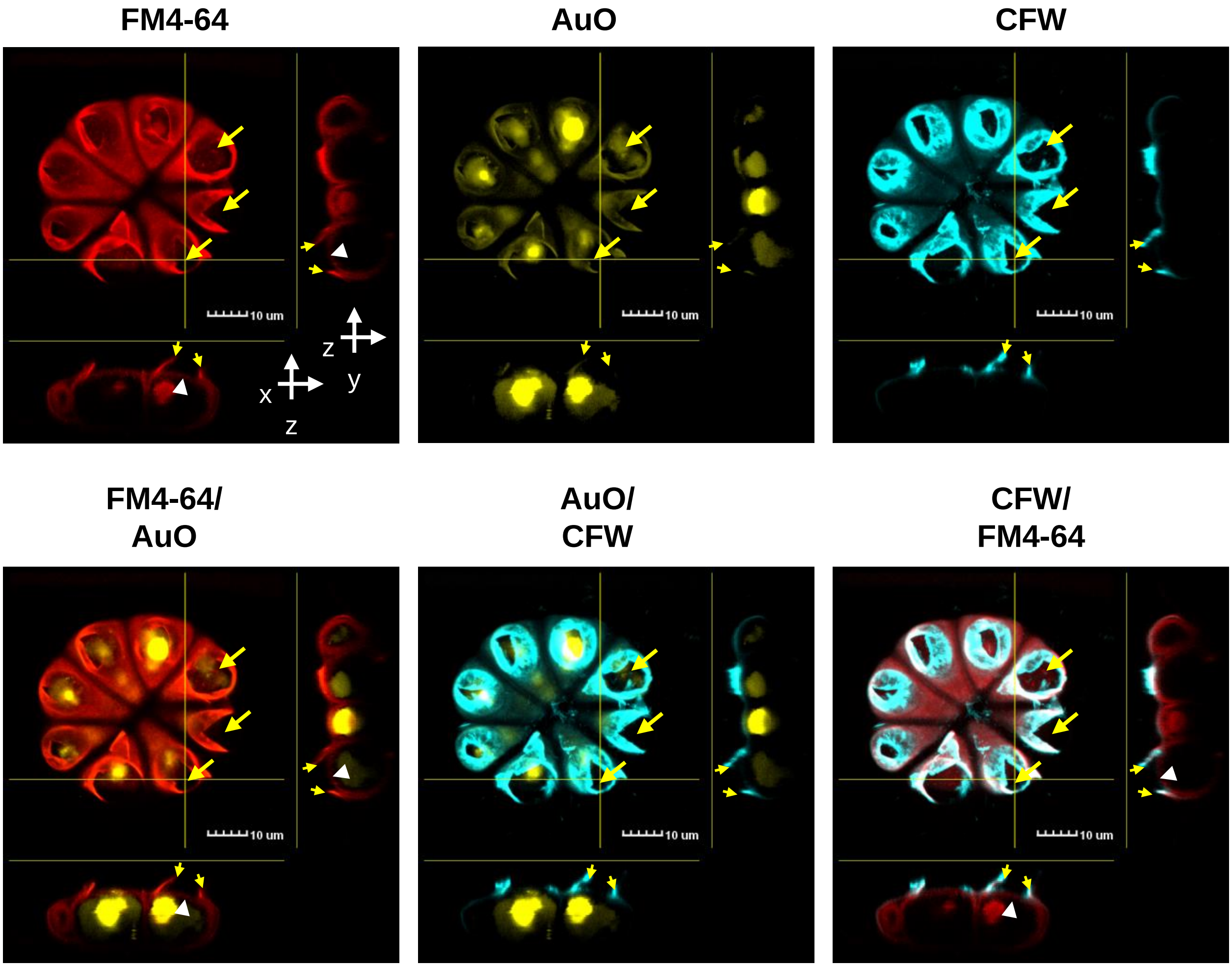

Figure S6. Analysis of the constituents of pores on the sessile trichomes. Leaf segments were co-incubated with FM4-64, AuO, and CFW and then displayed as z-projections to view the staining; 20 Z slices were acquired every 0.4 μm covering a total height of 8 μm. The side and bottom images for each picture show the longitudinal and the transverse sections, respectively, taken from sections indicated by the yellow lines. Yellow arrows indicate pores on cells. Small yellow arrows and white arrowheads indicate volcanic craters and round barriers detected by CFW/AuO/FM4-64 and FM4-64, respectively, around the discontinuous cell wall regions on the sessile trichomes. Scale bar = 10 μm.

Graphical abstract

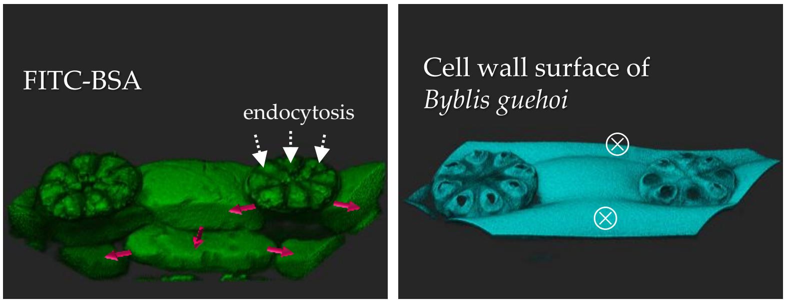

FITC-BSA, after endocytosed by sessile trichomes in *Byblis guehoi*, was transported to the short epidermis and then only downwards to mesophylls, but not the terminally differentiated epidermis, indicating a maximized efficiency in nutrient transport evolved in the carnivorous plant.
